# Tracing Cognitive Processes in Insight Problem Solving: Using GAMs and Change Point Analysis to Uncover Restructuring

**DOI:** 10.3390/jintelligence11050086

**Published:** 2023-05-03

**Authors:** Mario Graf, Amory H. Danek, Nemanja Vaci, Merim Bilalić

**Affiliations:** 1 Institute of Psychology, University of Klagenfurt, 9020 Klagenfurt, Austria; 2 Department of Psychology, Heidelberg University, 69117 Heidelberg, Germany; 3 Department of Psychology, Sheffield University, Sheffield S10 2BP, UK; 4 Department of Psychology, University of Northumbria at Newcastle, Newcastle upon Tyne NE1 8ST, UK

**Keywords:** insight problems, representational change, problem-solving, generalized additive models, change points analysis

## Abstract

Insight problems are likely to trigger an initial, incorrect mental representation, which needs to be restructured in order to find the solution. Despite the widespread theoretical assumption that this restructuring process happens suddenly, leading to the typical “Aha!” experience, the evidence is inconclusive. Among the reasons for this lack of clarity is that many measures of insight rely solely on the solvers’ subjective experience of the solution process. In our previous paper, we used matchstick arithmetic problems to demonstrate that it is possible to objectively trace problem-solving processes by combining eye movements with new analytical and statistical approaches. Specifically, we divided the problem-solving process into ten (relative) temporal phases to better capture possible small changes in problem representation. Here, we go a step further to demonstrate that classical statistical procedures, such as ANOVA, cannot capture sudden representational change processes, which are typical for insight problems. Only nonlinear statistical models, such as generalized additive (mixed) models (GAMs) and change points analysis, correctly identified the abrupt representational change. Additionally, we demonstrate that explicit hints reorient participants’ focus in a qualitatively different manner, changing the dynamics of restructuring in insight problem solving. While insight problems may indeed require a sudden restructuring of the initial mental representation, more sophisticated analytical and statistical approaches are necessary to uncover their true nature.

## 1. Introduction

In cognitive science, the temporal dynamics of problem-solving processes have always been an important topic of investigation. Most problems are assumed to be solved gradually, by piecing together information in order to arrive at a solution (Newell and Simon 1972). To investigate these problems, several tools have been developed, which allow for the observation of each step of the problem-solving process (e.g., Tower of Hanoi, Hobbits and Orcs problem). In the case of “insight problems”, the solution often comes seemingly out of nowhere (Duncker 1945), despite the problem appearing unsolvable just a moment earlier. To be solved, insight problems are thought to require a fundamental, sudden change in the way the problem is perceived, a process referred to as restructuring or representational change (Ohlsson 1992; Wertheimer 1925). The restructuring from the initial, incorrect mental representation to the correct one is the key component in modern theories such as representational change theory (RCT) (Knoblich et al. 1999; Ohlsson 1984, 1992, 2011).

Although the sudden nature of the underlying restructuring process is a main theoretical assumption about insight, the evidence for this claim is inconclusive. Ohlsson (1992) even hypothesized that “the sudden appearance of the complete solution in consciousness is an illusion caused by our lack of introspective access to our cognitive processes (...)” (p. 17). To truly understand the temporal nature of insight, the cognitive component of insight (restructuring) must be examined with appropriate tools. Observing changes in solvers’ mental problem representation is a methodological and statistical challenge, which is addressed in the present work. Among the reasons for this lack of clarity is that many measures of insight rely solely on the solvers’ subjective experience of the solution process. Using matchstick arithmetic problems, we demonstrate that it is possible to objectively trace problem-solving processes.

We first review the research on representational change, focusing on the experimental designs. After that, we describe a novel analytical approach that improves upon previous attempts. Finally, and arguably most importantly, we show that this analytical approach needs to be combined with appropriate statistical tools in order to work properly. We demonstrate the feasibility of this approach by re-analyzing eye-tracking data from an already published study (Bilalić et al. 2019). The paper is accompanied by an online supplement, with technical details, such as data and code for the analysis, which is freely available at https://osf.io/pwuhs/?view_only=7c52bda4e6fa481e826e5d7570b6ef3 (accessed on 25 April 2023).

### 1.1. Temporal Dynamics of the Restructuring Process

In 1994, Durso and colleagues conducted an early study on the temporal dynamics of insight problem solving. They asked participants to rate the relatedness of word pairs in a word puzzle and found that, on average, solution-relevant pairs were rated as increasingly similar as participants approached a solution. The authors concluded that “[l]ike dynamite, the insightful solution explodes on the solver’s cognitive landscape with breathtaking suddenness, but if one looks closely, a long fuse warns of the impending reorganization” (Durso et al. 1994, p. 98). Novick and Sherman (2003; Experiment 2) provided similar evidence. They asked participants to indicate within a short time window (250 ms after stimulus offset) whether presented anagrams were solvable. They found that, although participants could not find the solution within the allotted time, they were increasingly better at differentiating between solvable and unsolvable anagrams as the presentation time of the anagrams increased. The authors concluded that solvers gradually accumulate information relevant for solving the anagrams.

Several studies have focused on the concept of restructuring in insight problem solving, but have typically not measured the dynamics of the solving process (e.g., Ash et al. 2012; Ash and Wiley 2006, 2008; Fleck and Weisberg 2013; MacGregor and Cunningham 2009). However, a number of studies have attempted to measure the temporal dynamics of restructuring, using different methods to acquire trace data. Some used repeated ratings of problem elements, either regarding their similarity (Durso et al. 1994) or with regard to their relevance for the solution (Cushen and Wiley 2012; Danek et al. 2020). Others recorded eye movements (Ellis et al. 2011; Knoblich et al. 2001; Bilalić et al. 2019; Tseng et al. 2014) or employed solvability judgments (Novick and Sherman 2003). In some of these studies, both incremental and sudden solution patterns were found (Cushen and Wiley 2012; Danek et al. 2020; Novick and Sherman 2003), whereas other studies found only incremental patterns (Durso et al. 1994).

### 1.2. Eye Movements and Matchstick Arithmetic Problems

Here, we will take a closer look at using eye movement recordings to measure the temporal dynamics of restructuring in insight problems (for a comprehensive overview on eye movements, please see Holmqvist et al. 2011). In general, eye movements provide an objective measure of cognitive processes, as they are closely linked to attention (e.g., Just and Carpenter 1976; Rayner 1995; Reingold et al. 2001). Specifically, eye fixations reveal when people pay attention to certain features of a problem and for how long. More importantly, eye tracking is particularly useful when participants might not remember or even concurrently report that they are paying attention to these elements (Bilalić and McLeod 2014; Bilalić et al. 2008, 2010; Kuhn and Land 2006; Kuhn et al. 2009). This is particularly relevant in the case of insight problems, where it is possible that people are not aware of the dynamics of their solution process.

**Figure 1:**
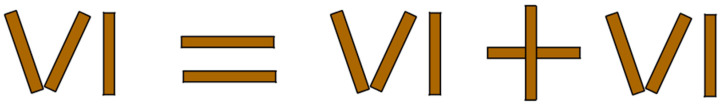
Matchstick arithmetic problem. Participants are required to transform the false arithmetic statement to a true statement by moving a single matchstick. This problem requires restructuring, because the initial assumption that only the matchsticks from values can be manipulated needs to be changed. In this case, the operator “+” can be decomposed and its vertical matchstick moved to make another “=” sign (VI = VI = VI). The “+” sign is the critical element that needs to be changed for solution.

We use the matchstick arithmetic problems introduced by Knoblich et al. (1999). Matchstick arithmetic problems are suitable for investigation with eye tracking, as was powerfully demonstrated by the seminal study of Knoblich et al. (2001). A matchstick arithmetic problem consists of a false arithmetic statement written using Roman numerals, arithmetic operators, and equal signs, all formed using matchsticks (Knoblich et al. 1999, 2001; see also Figure 1 below). The task is to transform the false arithmetic statement into a true statement by moving only a single stick. Four types of matchstick arithmetic problems have been defined with varying levels of difficulty, depending on the constraints that need to be relaxed and the tightness of the chunks that need to be decomposed. These problem types were theoretically derived from the representational change theory (Ohlsson 1992) and have been empirically confirmed (Knoblich et al. 1999; Öllinger et al. 2006, 2008). The use of matchstick arithmetic problems enables us to build on a well-researched task domain. It is known which problem type should elicit the restructuring process (Knoblich et al. 1999; Öllinger et al. 2006, 2008), and it is possible to contrast it with a type which requires no restructuring. Additionally, based on Knoblich’s study (2001), predictions about eye movement patterns can be made. Furthermore, the matchstick arithmetic domain is well suited for eye tracking because each problem consists of individual matchsticks that do not overlap, allowing for precise differentiation of fixations. In other words, we can determine at any point in time which aspect of the problem is attended to.

Knoblich et al. (2001) investigated constraint relaxation type problems, which are considered to require restructuring; see Figure 1 for an example. They found that for this problem (constraint relaxation type), both solvers and non-solvers examined the values in the beginning and spent most of their time doing so. This can be seen as an indication that participants were using an initial incorrect problem representation, triggered by previous knowledge, where only values can be changed. Only in the final third of the problem- solving period did later solvers change their mental representation, as demonstrated by their eye movements. Solvers started to pay attention more to the operators and less to the values. In contrast, non-solvers remained stuck in their initial representation, as they continued to attend to values rather than to operators. Similar results for the same problem were found by another eye-tracking study (Tseng et al. 2014).

The Knoblich et al. (2001) study provides strong evidence for the claim that in problems that require constraint relaxation, a restructuring of the problem representation took place. However, it did not answer the question of whether this change was a sudden or a gradual one. In the final third of the allotted time, solvers paid attention to the important but previously ignored features, which could be interpreted as a result of sudden restructuring. It is nevertheless not that clear, since the final period may have lasted minutes, given that they took around five minutes to solve the problem. Thus, the restructuring might have been a continuous process over time. On the other hand, an eye-tracking study on anagrams by Ellis et al. (2011; see also Ellis and Reingold 2014) found that participants started disregarding the irrelevant problem elements several seconds before they came up with the solution. The viewing times on that problem elements were decreasing gradually. Most intriguingly, both participant groups, those who experienced pop-out insight-like solutions and those who did not, displayed the same gradual accumulation of solution knowledge.

### 1.3. Metacognitive Processes and Insight Problems

There is evidence that the problem-solving process benefits from hints (e.g., Bowden 1997; Bilalić et al. 2019; Ammalainen and Moroshkina 2021; Becker et al. 2021; Korovkin and Savinova 2021; Spiridonov et al. 2021). This is the case even when hints were unreportable; that is, hints even work when presented briefly below the threshold of consciousness. Ammalainen and Moroshkina (2021) found evidence that hints can influence the problem-solving ability, which can be both, positive and negative. In a positive way, hints which are helpful to find the solution increase solution rates. On the other hand, misleading hints can negatively affect solution rates by distracting problem solvers and leading to a decrease in their success rate. In our paper (Bilalić et al. 2019), we also provided hints when participants were unable to find the correct solution after a certain time.

These hints serve two purposes: a practical and a theoretical one. On a practical level, they provide an additional check on the main assumption behind the restructuring process. On a theoretical level, they serve as explicit clues that tap into metacognitive processes (Takeuchi et al. 2019; Metcalfe and Shimamura 1994). Hints make participants aware of important aspects in the problem, drawing their attention towards elements that may have been neglected. They also change participants’ knowledge about the problem, potentially affecting the way they solve insight problems (Bowden 1997; Bilalić et al. 2019; Korovkin and Savinova 2021).

## 2. Methods

The present work is a re-analysis of our paper (Bilalić et al. 2019). In our paper, we also combined solving of insight and non-insight problems with eye tracking. We presented first a non-insight matchstick problem and then the matchstick insight problem depicted here (see Figure 1) to 61 participants (5 male; *M*_age_ = 22.8; *SD*_age_ = 6.5). The study was designed to take into account the methodological issue discussed in the previous section. It built upon previous attempts that utilized more time periods and sometimes presented the last 5 or 10 s separately (see also Bilalić et al. 2008, 2010, 2014). In the 2019 study, we provided a more fine-grained temporal analysis of the solution process by using ten time periods of equal length for our eye movement analysis^1^ (for more information, please refer to Bilalić et al. 2019). We demonstrated that the restructuring is a gradual process on the insight problem as the solvers started paying attention to the important aspects of the problem long before they found the solution. Here, we provide another set of data where the jump is sudden; that is, the solvers started paying attention to the important aspects immediately before they found the solution (as reported by Knoblich et al. 2001). This is done to illustrate (1) how classical ways of analyzing data, such as ANOVA, are inappropriate for discovering the sudden changes, and (2) how other non-linear approaches are required.

We expected that all participants would initially focus on the values. Solvers would shift their attention towards the critical element (the “+” operator), while non-solvers would remain fixated on the values. The first question of interest is whether the representational shift in eventual solvers will be sudden or rather incremental. The second question of interest is whether the explicit cue, that is, the hint, will produce a sudden rearrangement of attention towards the critical elements (here “+”, but also “=” because “=” is also an operator). In our design, we included hints for participants who had not solved the problem within five minutes. The hint provided at this point was ‘You can change the operators, too.’ We were interested in whether the hints change the dynamics of problem solving, specifically whether the solution process remains sudden even after receiving an explicit cue.

## 3. Results

The problem proved difficult as only 34% found the solution. After the hint was provided, an additional 11% of participants were able to find the solution. We present the eye data analysis below, with a particular focus on the critical element of the problem, the plus sign (+). Additionally, when analyzing the impact of hints, we also focused on the equal sign (=) as the hints should also affect the attention drawn to this operator through metacognitive control. For analysis of other problem elements, please refer to the supplementary materials.

### 3.1. Is Insight Sudden or Incremental? (Solvers vs. Non-Solvers: First 5 Min Analysis)

**Figure 2:**
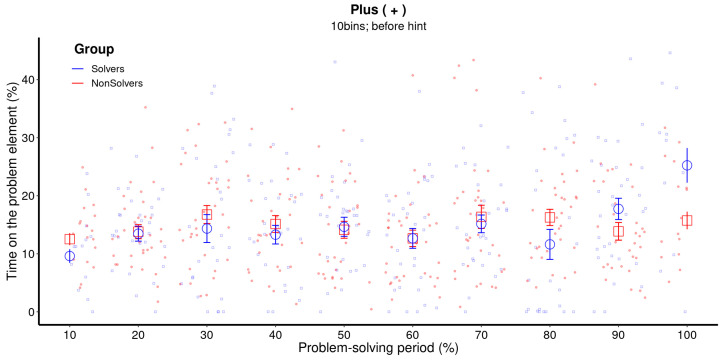
**Raw data and means for each bin of the critical element (+).** The raw data represents every data point of each participant over the entire problem-solving period. The problem-solving period was divided in 10 proportional bins, each representing 10% of the total problem-solving time. The error bars represent the 68% confidence interval. This figure illustrates a nonlinear increase in the amount of time that solvers spend on the critical element. In the case of solvers, the 100% bin means the participant provided a solution.

In Figure 2, raw data and means for each bin of the critical element for the first five minutes are presented.^2^ The solving pattern follows the typical sudden pattern, where there is not much difference between eventual solvers and non-solvers with regard to the time spent on the critical element (+) until the end of the first five minutes. Solvers suddenly increase their dwell time just before announcing the solution, while non-solvers continue to observe the critical element sporadically until the end of the solving period.

**Figure 3:**
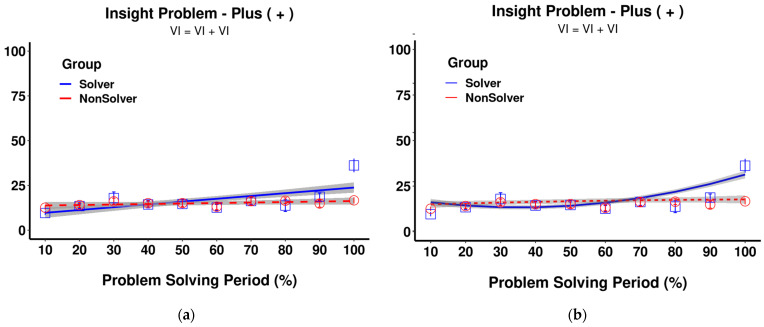
Estimated model means based on (**a**) ANOVA with linear term; (**b**) ANOVA with both linear and quadratic terms. Y-Axis: Time on the problem element (%). Please refer to supplementary material for the detailed analysis.

The crucial question is how to analyze the temporal changes presented in Figure 2. The traditional method, which we had chosen in our previous paper (Bilalić et al. 2019), is to use an analysis of variance (ANOVA) where the bins and groups are factors that predict the amount of time spent on the critical element. However, ANOVA not only requires a completely balanced dataset, but it also ignores the clustered nature of data (van Rij et al. 2020). Furthermore, it is based on linear regression, which is not suitable for capturing sudden attentional shifts, which are nonlinear in nature. In order to capture the sudden shift as depicted in Figure 2 (the 100% bin for the solvers), ANOVA would need to adjust the linear trend throughout the whole problem-solving period. In other words, a sudden trend may appear as an incremental one as ANOVA adjusts by increasing previous periods (see Figure 3, left panel).

ANOVA can be expressed as linear regression, where an additional quadratic polynomial term is included next to the linear one, in an attempt to capture the shift. However, even in this case, the predicted shift by the ANOVA model would begin earlier, namely at the 80% bin, than it does in the raw data (see Figure 3, right panel). The general limitation of linear regression, with or without polynomial terms, is that it heavily relies on previous trends. If the change is sudden, the previous time periods will also be adjusted accordingly.

One way around this problem is generalized additive (mixed) modeling (GAM). These models are specifically designed to handle nonlinear relationships, as they are data-driven and use non-linear mixed-effects regression (van Rij et al. 2020). A key benefit of GAMs is that they do not require the user to specify the shape of the nonlinear regression line, as the model determines this based on the data. However, while GAMs have a high level of flexibility in modeling nonlinear changes in time series data, they only allow for the exploration of changes in the function and do not provide parametric estimates such as standard error of estimate or its impact on predictive accuracy of the model. More specifically, GAMs do not provide parametric estimates, which means that they do not give us a set of parameters that describe the shape of the nonlinear function. However, the present work intends to demonstrate the advantages and downsides of the available analysis tools in question, which is why GAMs are included here.

Arguably the most reliable way of checking the assumption of suddenness is the use of change point analysis, which looks for significant deviance from previous trends (Raftery and Akman 1986). Unlike the standard regression analysis (ANOVA) and nonlinear GAMs, change point regression estimates the moment of the function inflection. In other words, it includes the possibility to estimate additional parameters, such as intercept and slope of regression, time point when the function changes, and how the intercept and/or slope of regression changes (see the figures of the MCP analysis for illustrations). This makes the technique particularly valuable in detecting increasing patterns as one would expect several points of change in the attentional pattern on the way towards the solution. In this instance, we use the one implemented in the Multiple Change Points package (MCP; Lindeløv 2020).

**Figure 4:**
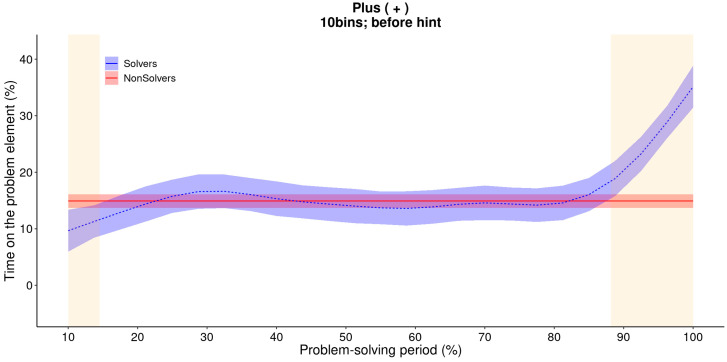
**GAM: the difference between two estimated trend lines for solvers and non-solvers of the critical element (+).** This figure illustrates that the GAM also found a nonlinear increase in the amount of time that non-solvers spend on the critical element. The orange area determines where the differences between solvers and non-solvers were significant.

Below, we address the three main questions using both GAM and MCP analysis. In the supplemental material, we provide the model-estimated values for each case, which include the results and, in the case of the MCPs, how well the model fits the data and which model was used. We begin with the GAM analysis of solvers and non-solvers for the first five minutes to determine whether the insight is sudden or incremental. Figure 4 provides the estimated trend lines for both solvers and non-solvers, as well as the time periods (shaded in orange) where the difference between the two is statistically significant. The model estimates closely follow the raw data (see Figure 2), and the difference between solvers and non-solvers is indeed significant at the beginning of the solving phase, as well as at the 90% bin and the 100% bin.

**Figure 5:**
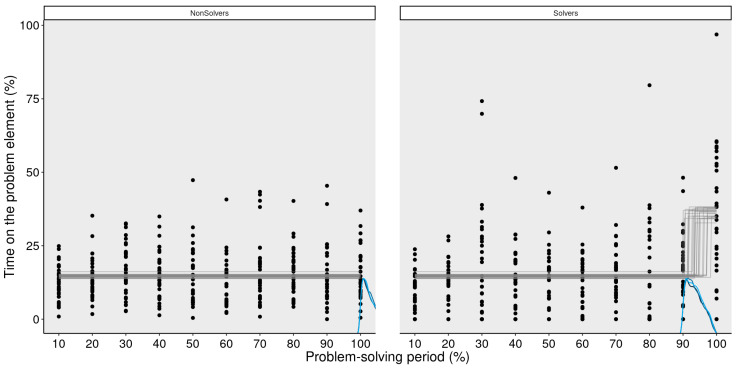
**MCP analysis of the critical element (+) for non-solvers and solvers.** This figure illustrates every data point of each participant over the problem-solving period. Lines at the bottom of the figure illustrate the posterior density (estimated likelihood) of the change point for each MCMC chain. There is a nonlinear increase in the amount of time that solvers spend on the critical element.

Figure 5 shows the results of the MCP analysis for the same data as the GAM above. Similarly to the GAM, the MCP analysis identified a change point around the 90% bin for the solvers, which captures an attentional shift they made. While some non-solvers also shifted their attention towards the “+” sign at the end, it was not as clear as in the case of the solvers.

### 3.2. Do Explicit Cues Rearrange Attentional Distribution? (An Immediate Change after the Hint)

**Figure 6:**
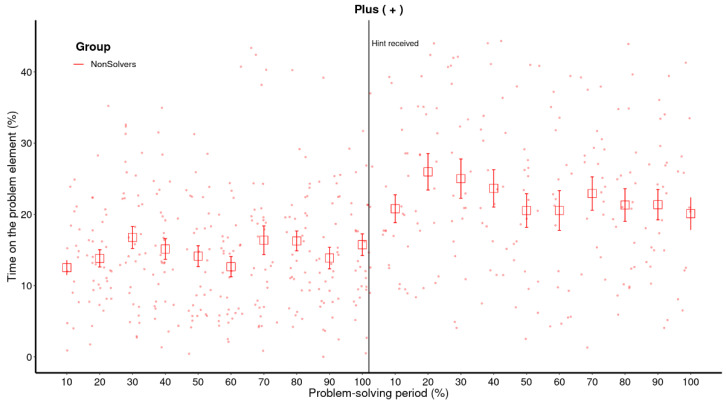

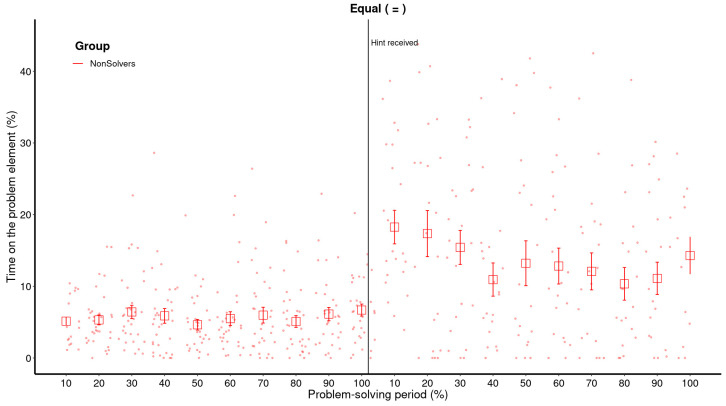
**Raw data and means for each bin of the operators (“+” upper panel; “=” lower panel) in the period before and after the hint.** The raw data represents every data point of each participant (non-solvers only) over the problem-solving periods. Each of both problem-solving periods (before and after the hint was provided) were divided in 10 proportional bins, each representing 10% of the total problem-solving time. It is necessary to view the problem-solving periods as distinct periods; therefore, each period is labeled from beginning to end (10% to 100%) to differentiate them. The error bars represent the 68% confidence interval. This figure illustrates the attentional shifts from values (mostly attended to before the hint) towards operators (attended to after the hint was given).

Figure 6 illustrates the impact of providing an explicit hint to the non-solvers from the first five minutes (presented here as a single group; solvers from the first five minutes are not included in this graph). The attentional shift from values towards operators, “+” and “=”, is substantial immediately after the hint. The operator “=” is attended to twice as much immediately after the hint than before. The change for “+” is slightly less dramatic at first (only 4%), but by the 20% bin, the dwell time has doubled in comparison to before the hint was provided. Note that only non-solvers are shown here, since solvers did not receive any hints.

**Figure 7:**
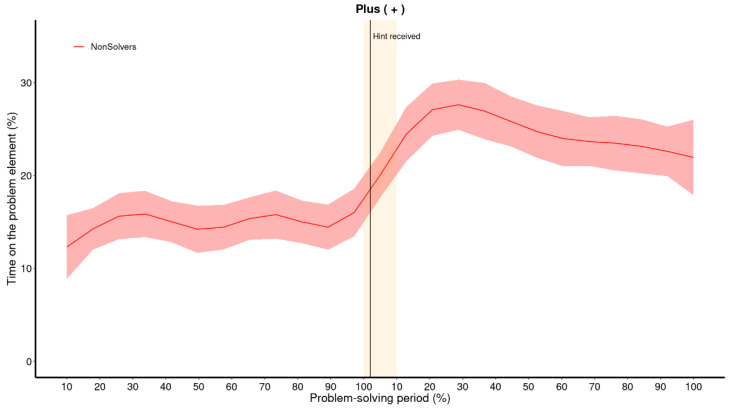

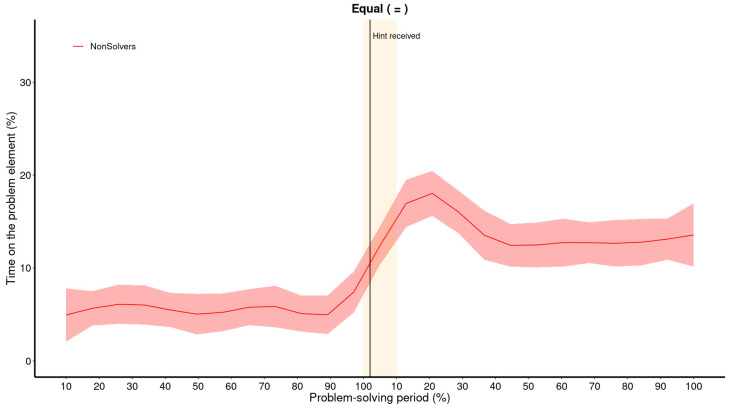
GAM: estimated trend line for non-solvers of the critical element (+; upper panel) and the other operator (=; lower panel). This figure illustrates that the GAM also found a nonlinear increase in the amount of time that non-solvers spend on the critical element after receiving a hint. The orange area indicates where there is a significant shift in attention.

The GAM analysis effectively captures the attentional shift, as depicted in Figure 7. However, it predicts that the change occurs prior to the hint being provided, starting already at the 90% bin, which is not a correct reflection of the actual data. While GAM is considerably more flexible than regressions with polynomial terms, the same problem of interdependence of neighboring phases remains. The shift caused by the explicit cue is so drastic that the GAM needs to adjust the increase to begin earlier in order to account for it.

**Figure 8:**
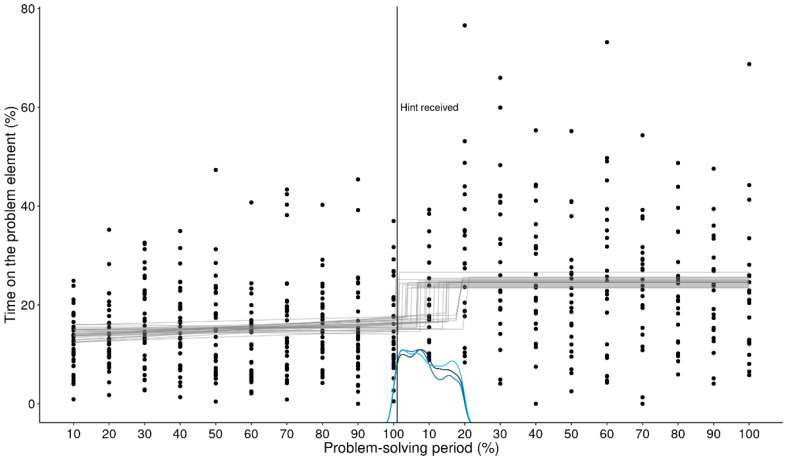

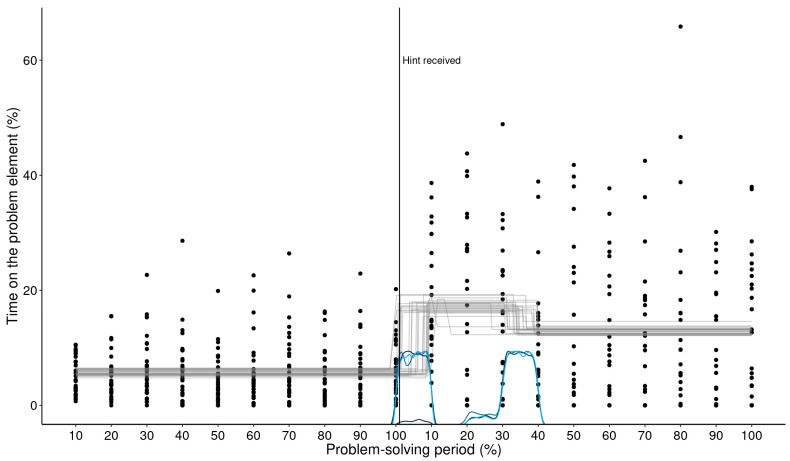
**MCP analysis of the critical element (+; upper panel) and the other operator (=; lower panel).** This figure illustrates that the switch points of the MCP analysis correctly captures where the shift in attention happens.

In contrast, the switch points of the MCP analysis correctly capture where the change in attention allocation happens (see Figure 8).

### 3.3. Does Metacognition Influence Insight Problem Solving? (Solvers vs. Non-Solvers after the Hint)

**Figure 9:**
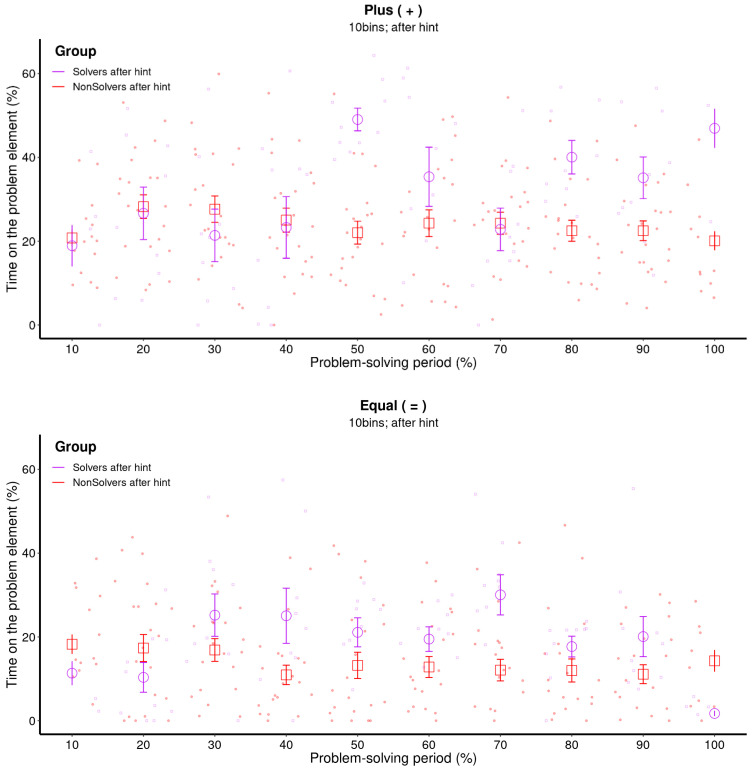
**Raw data and means for each bin of the critical element (+; upper panel) and the other operator (=; lower panel) after the hint was provided.** The raw data represent every data point of each participant over the remaining problem-solving period after the hint was given. The error bars represent the 68% confidence interval. This figure illustrates a nonlinear increase or decrease in the time solvers spend on the critical element.

The final question we aimed to address was whether the explicit cue, and the additional knowledge about the problem associated with it, would alter the way the problem was solved. Figure 9 indicates that both solvers and non-solvers maintain the level of attention on the critical aspects throughout the problem-solving period, which is a direct consequence of the explicit cue. However, this was not sufficient for finding the solution. The eventual solvers initially shifted their attention to “=” around the 30% bin, but starting from the 50% bin, they increasingly focused on “+”. This means that at this point in time, the solvers may have realized that the “+” symbol was the critical element they needed to solve the problem. Consequently, they gathered more information about the symbol by attending to it more closely.

**Figure 10:**
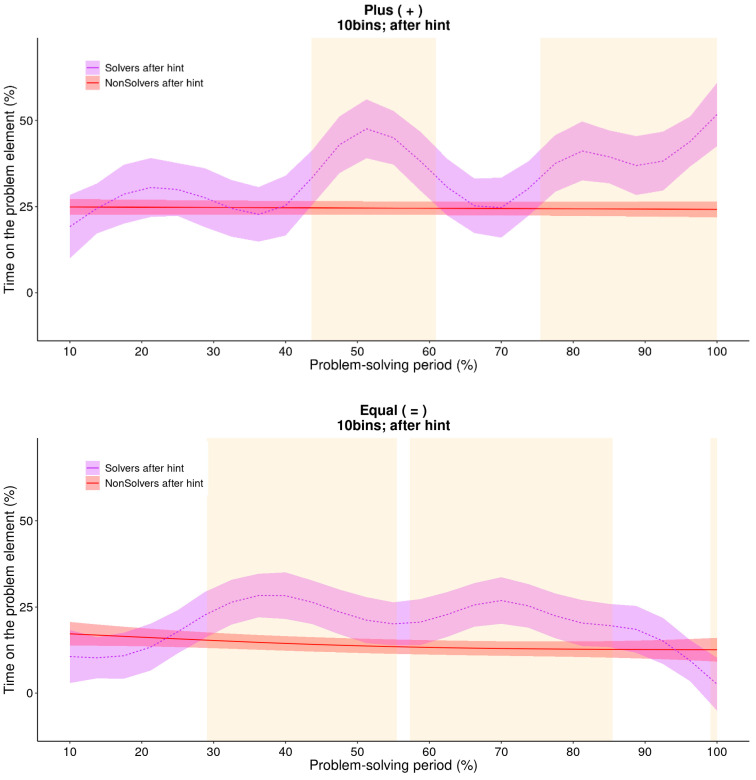
**GAM: the difference between two estimated trend lines for solvers and non-solvers of the critical element (+; upper panel) and the other operator (=; lower panel).** This figure illustrates that the GAM also found a nonlinear increase in the time solvers spend on that particular element. The orange area in the figure indicates regions where there are statistically significant differences between the attention patterns of solvers and non-solvers.

This incremental pattern of solving is well captured by GAMs, as Figure 10 illustrates. While the non-solvers attended to the critical “+” operator consistently over the entire problem-solving period, but on a rather low level of 25% of their time, solvers gradually increased their attention towards it. Significant differences were found in the middle and the end of the problem-solving period. This was also the case for the other operator (=). Non-solvers attended to “=” in a consistent manner throughout the problem-solving period, while the solvers attended to “=” more in the middle of the problem-solving period and less at the very end of it, probably because they were then already focusing more on the “+” sign which needs to be changed for a solution.

**Figure 11:**
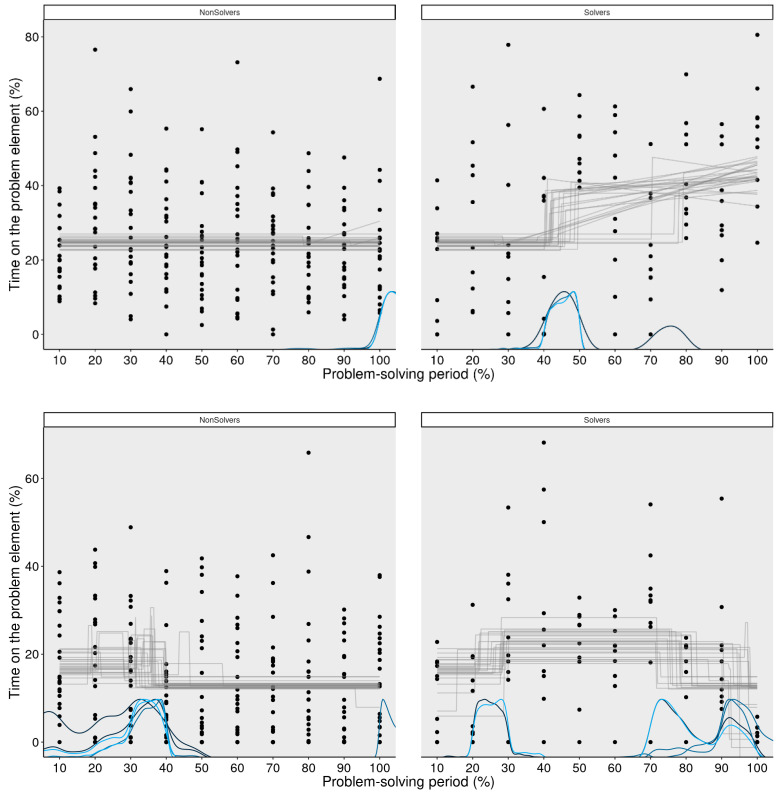
**MCP analysis of the critical element after the hint (+; upper panel) and the other operator (=; lower panel) for non-solvers and solvers.** This figure demonstrates that the switch points of the MCP analysis correctly captures the incremental pattern of solving for the critical element (+). It also demonstrates that after the hint, the non-solvers attended to the noncritical element (=) more in the beginning but not the critical element (+). As the GAMs showed already, the non-solvers attended to the critical element (+) in the same way throughout the whole problem-solving period.

Figure 11 illustrates that the attentional shifts after receiving a hint are effectively captured by the MCP analysis. Again, non-solvers attended to the “+” operator on a consistently low level throughout the entire problem-solving process, while solvers attended to the “+” operator more and more. The same trend is observed for the “=” operator. Non-solvers attended to it less, while solvers shifted their attention to it in the middle of the problem-solving process. Towards the end of the problem-solving process, the data suggest that solvers became aware that the “=” operator was not as important for solving the problem and began to focus more on the “+” operator.

## 4. Discussion

We have demonstrated that recording eye movements is a valuable method for gaining insight into complex cognitive processes, including mental restructuring in insight problems. It is also an adequate tool for investigating attentional shifts after receiving hints. However, it is important to use eye movement recording with appropriate analytical approaches. Our results show that it is necessary to conduct a more fine-grained analysis of the eye movement data to capture the temporal dynamics of the problem-solving process. This is particularly relevant for insight problems such as the one used here, which are believed to feature a sudden change in eye movement patterns reflecting a change in mental representation.

We were able to identify the point at which solvers and non-solvers start to differ in their attentional patterns by dividing the problem-solving period into ten equal bins. The temporal resolution of the problem-solving period is one aspect, but it is also important to choose an appropriate statistical procedure. We have demonstrated that nonlinear statistical models, such as GAM and MCP, can effectively capture the sudden change that is a hallmark of insight problem solving. The GAM analysis can effectively capture the attentional shift; however, it predicts that the change occurs prior to the correct reflection of the actual data. While GAM is considerably more flexible than regressions with polynomial terms, the same problem of interdependence of neighboring phases remains. The shift caused by the explicit cue is so drastic that the GAM needs to adjust the increase to begin earlier to account for it. In contrast, the change points of the MCP analysis correctly capture where the change in attention allocation happens. A change point is a time point where the statistical properties of a time series change abruptly. However, in contrast to GAMs, one needs a priori knowledge about the number of change points and the form of the segments in between (Lindeløv 2020). Therefore, one might decide from case to case which statistical procedure is appropriate.

Our example illustrates the importance of considering theoretical assumptions when choosing analytical and statistical procedures. The restructuring of mental representations is a key concept in theories of insight (Knoblich et al. 1999; Ohlsson 1984, 1992, 2011). It is a nonlinear process in essence, which can be operationalized as a sudden burst of attention to the relevant aspects of a problem (Bilalić et al. 2019). The shift inevitably deviates significantly from participants’ previous problem solving. Seen as a part of the overall problem-solving continuum, the sudden shift is difficult to capture with linear statistical procedures. Only truly nonlinear statistical procedures can appropriately capture the sudden nature of representational change.

Providing explicit hints typically alters the dynamics of problem solving. It is obvious that the given hints were effective, as participants’ patterns of attention show a drastic change, which is very well captured by both GAM and MCP. However, it is important to note that the eventual solvers, after receiving the hint, exhibit a gradual, incremental shift, with increasing attention to the main elements during the problem-solving period. In contrast, non-solvers display an immediate burst of refocusing following the hint, but subsequently, their attention to the important aspects diminishes.

Both the analytical procedure for capturing the temporal resolution and the nonlinear statistical procedures can be easily extended beyond eye movements to other tracing methods. For example, “importance-to-solution” ratings of individual problem elements that are made repeatedly during the solving process (Durso et al. 1994; Cushen and Wiley 2012; Danek et al. 2020; Danek and Wiley 2020) often reveal patterns of sudden change which could be effectively captured by GAMs and MCPs. Similarly, “Feelings-of-Warmth” that are used to assess metacognitive knowledge about solution progress (Kizilirmak et al. 2018; Hedne et al. 2016; Pétervári and Danek 2020) are another suitable candidate for nonlinear modeling with GAMs. Other tracing methods, such as mouse-tracing data (Loesche et al. 2018; van Rij et al. 2020), think-aloud protocols (Gilhooly et al. 2010; Schooler et al. 1993; Blech et al. 2020), or even self-reports (Fedor et al. 2015), are also better modeled with GAMs than with commonly applied linear methods, even if they are more appropriate than the classical ANOVA.

## 5. Conclusions

Our results indicate that for insight problems, the restructuring process leaves a discernible trace of suddenness. Eye movements suggest that just prior to solving the problems, participants shift their focus from elements that constituted the initial problem representation to those crucial for the solution. Our results also demonstrate that receiving hints leads to attentional shifts towards critical aspects, which in turn facilitates the generation of a correct solution. However, in order to accurately capture the sudden shift in attention, a combination of the appropriate methodological approach and statistical procedure is necessary. These nonlinear processes are best captured by nonlinear statistical procedures, such as GAMs and MCPs.

## Data Availability

Technical details, such as data and code for the analysis, is available at https://osf.io/pwuhs/?view_only=7c52bda4e6fa481e826e5d7570b6ef34 (accessed on 25 April 2023).

## Notes

1 The length of time taken to solve (or not solve) a problem is different from person to person, meaning that one cannot compare the eye tracking data directly between people. For example, some may need only 45 s to solve the problem, whereas others need four minutes to find a solution. In consequence, the data must be transformed in order to be able to compare the data between people properly. While the problem-solving period can be extended by adding more time phases, it is important to note that the duration should not be prolonged beyond a certain point. Utilizing too many time frames may leave too little data (e.g., a 10-second trial should not be divided into 100 bins, as each bin will have the duration of only 100 ms). This can lead to distorted eye movement patterns, masking the underlying effects present before the data were binned. On the other hand, choosing too few bins may not capture the full temporal dynamics of the problem-solving process. In either case, ANOVA is not suitable for analyzing a large number of problem-solving periods, unlike GAM and multiple change point analysis, which can easily accommodate a large number of time frames. MCP analysis is another adequate tool for this type of analysis as it can capture the shift of attention. However, in contrast to GAMs, one needs a priori knowledge about the number of change points and the form of the segments in between (Lindeløv 2020).

2 Please note that the data presented here are simulated to represent a sudden shift, which is difficult to capture by classical analyses. The original data in Bilalić et al. (2019) indicate a gradual shift.

## Supplementary Materials

The following supporting information can be downloaded at: https://osf.io/pwuhs/?view_only=7c52bda4e6fa481e826e5d7570b6ef34.
